# Metabolic reprogramming of *Salmonella* infected macrophages and its modulation by iron availability and the mTOR pathway

**DOI:** 10.15698/mic2019.12.700

**Published:** 2019-11-14

**Authors:** Julia Telser, Chiara Volani, Richard Hilbe, Markus Seifert, Natascha Brigo, Giuseppe Paglia, Günter Weiss

**Affiliations:** 1 Department of Internal Medicine II, Medical University of Innsbruck, Austria.; 2 Christian Doppler Laboratory for Iron Metabolism and Anemia Research, Medical University of Innsbruck, Austria.; 3 EURAC Research, Institute for Biomedicine, Bolzano/Bozen, Italy.; 4 School of Medicine and Surgery, Univerity of Milano-Bicocca.

**Keywords:** macrophage, Salmonella, Krebs cycle, iron, mTOR, glycolysis

## Abstract

Iron is an essential nutrient for immune cells and microbes, therefore the control of its homeostasis plays a decisive role for infections. Moreover, iron affects metabolic pathways by modulating the translational expression of the key tricarboxylic acid cycle (TCA) enzyme mitochondrial aconitase and the energy formation by mitochondria. Recent data provide evidence for metabolic re-programming of immune cells including macrophages during infection which is centrally controlled by mTOR. We herein studied the effects of iron perturbations on metabolic profiles in macrophages upon infection with the intracellular bacterium *Salmonella* enterica serovar Typhimurium and analysed for a link to the mTOR pathway. Infection of the murine macrophage cell line RAW264.7 with *Salmonella* resulted in the induction of mTOR activity, anaerobic glycolysis and inhibition of the TCA activity as reflected by reduced pyruvate and increased lactate levels. In contrast, iron supplementation to macrophages not only affected the mRNA expression of TCA and glycolytic enzymes but also resulted in metabolic reprogramming with increased pyruvate accumulation and reduced lactate levels apart from modulating the concentrations of several other metabolites. While mTOR slightly affected cellular iron homeostasis in infected macrophages, mTOR inhibition by rapamycin resulted in a significant growth promotion of bacteria. Importantly, iron further increased bacterial numbers in rapamycin treated macrophages, however, the metabolic profiles induced by iron in the presence or absence of mTOR activity differed in several aspects. Our data indicate, that iron not only serves as a bacterial nutrient but also acts as a metabolic modulator of the TCA cycle, partly reversing the Warburg effect and resulting in a pathogen friendly nutritional environment.

## INTRODUCTION

Iron is a crucial element for many organisms since it is a co-factor for enzymes and proteins involved in mitochondrial electron transport, citric acid cycle, metabolism, DNA synthesis and oxygen binding [1]. While iron deficiency negatively affects cellular proliferation and metabolic activity, iron overload results in cellular damage via iron mediated formation of toxic radicals by the Fenton reaction [2]. Thus, tight control of iron homeostasis is fundamental for proper cellular function. Iron is acquired by cells via different mechanisms including uptake of transferrin bound iron via transferrin receptor (TfR) mediated endocytosis, divalent metal transporter-1 (DMT-1) mediated ferrous iron uptake or in case of macrophages, receptor mediated incorporation of senescent erythrocytes. Intracellular iron is then either stored within the protein ferritin for future utilization, incorporated into iron containing proteins or exported from the cell by the transmembrane protein ferroportin [1, 3, 4]. The orchestration of those cellular iron genes is maintained by iron sensitive intracellular proteins, termed iron regulatory proteins (IRP)-1 and 2 which control the post-transcriptional and translational expression of these iron metabolism genes following binding to specific RNA stem loop structures, iron responsive elements (IREs), located within the 5' or 3' untranslated regions within the mRNA of the respective genes [1, 5].

During iron deficiency IRPs efficiently bind to IREs resulting in stabilization of TfR1 and DMT1 mRNA and consequently increased iron uptake whereas the translation of ferritin and ferroportin mRNA is blocked thus ensuring increased intracellular iron availability [1, 4]. If iron is abundant within cells, IRP1 acts as a cytoplasmic aconitase with no IRE binding affinity whereas IRP2 undergoes ubiquitylation and proteasomal degradation by the F-box and leucine-rich repeat protein 5 (FBXL5), an E3 ubiquitin ligase subunit [5, 6]. This results in ferritin and ferroportin translation with consecutive promotion of either iron storage or cellular iron export whereas iron uptake via TfR or DMT1 is reduced.

Of note, in its non-IRE binding form IRP-1 acts as a cytoplasmic aconitase with currently unknown physiological function whereas the translation of mitochondrial aconitase is controlled by IRE/IRP interaction in a way that surplus iron promotes mitochondrial aconitase expression whereas iron deficiency results in repression of mitochondrial aconitase translation [7, 8]. Mitochondrial aconitase is a central enzyme of the Krebs or tricarboxylic acid cycle (TCA), and modulation of cellular iron availability *in vitro* alters TCA enzyme activities, NADH formation, mitochondrial respiration and cellular oxygen consumption [9]. In rats iron deficiency had little effects on TCA activity but resulted in a significant decrease of citrate levels after three weeks [10], whereas in mice exposed to high dietary iron reprogramming of the Krebs cycle and altered glucose homeostasis was observed over time [11]. Of note, sustained iron loading had a negative effect on mitochondrial function via promotion of oxidative stress [12].

At the systemic levels iron homeostasis is controlled by the liver derived hormone hepcidin. Iron loading or inflammatory signals including lipopolysaccharide result in hepcidin induction and release to the circulation whereas iron deficiency or hypoxia block hepcidin expression [13]. Hepcidin exerts its regulatory function upon binding to ferroportin resulting in its internalization and degradation thereby blocking cellular iron egress from macrophages and enterocytes. Conversely, suppression of hepcidin expression leads to enhanced cell surface ferroportin expression and increased cellular iron release [14, 15].

The control over iron homeostasis appears to be crucial for the course of infections. This is because iron on the one hand is an essential growth factor for most microbes, and because the expression of microbial iron acquisition systems is linked to microbial pathogenicity [16, 17]. Moreover, iron exerts subtle effects on cellular immune regulation by affecting the differentiation of lymphocytes [18] but also by impacting on macrophage anti-microbial immune effector mechanisms including the formation of oxygen and nitrogen radicals, tumor necrosis factor (TNF) alpha or interleukin (IL) 1, 6 or 10 [19].

Of note, iron metabolism undergoes massive, inflammation driven chances during the course of an infection aiming at reducing the microbial access to this essential nutrient [15], and the specific mechanisms appear to be different according to the nature and localization of the respective pathogen [20–22]. Therefore, regulation of iron homeostasis by the host is inevitable in host-pathogen interaction and acts as a control mechanism against invading pathogens [16, 17, 22].

In addition, recent investigations reported a metabolic reprogramming in the course of infection. This metabolic change is characterized by a shift from oxidative phosphorylation towards anaerobic glycolysis [23, 24]. Energy is then mainly produced via glycolysis resulting in the accumulation of lactic acid even when enough oxygen is abundant [23, 25].

Mechanistically, part of this metabolic reprogramming is controlled by the mammalian target of rapamycin (mTOR) signaling pathway [25], and inhibition of the mTOR pathway negatively impacts the immune control intra-macrophage infection with *Mycobacterium tuberculosis* [26]. Of note, mTOR affects iron homeostasis by controlling hepcidin expression and TfR stability [27, 28]. The latter can be referred to tristetraprolin (TTP), which is a downstream target of mTOR [28]. Under iron-deficient conditions, this protein becomes activated which results in degradation of mRNAs of non-essential iron containing proteins, thereby liberating iron which can be used in vital processes. Moreover, TTP has the property to interact with TfR1 and to alter its stability which results in the degradation of the iron importer and changes in cellular iron flux [28–30].

Based on this evidence, we questioned whether the growth-promoting effect of iron on intramacrophage microbes such as *Salmonella* enterica serovar typhimurim [31], which will be also simply referred as *Salmonella* Tm, and the deterimental role of excess iron in bacterial sepsis [32] are not only due to the metal function as a microbial nutrient but also linked to iron mediated re-programming of macrophage energy metabolism via its effects on TCA and/or via modulation of mTOR activity.

## RESULTS

### Iron status regulates the metabolic reprogramming within macrophages.

**Figure 1:**
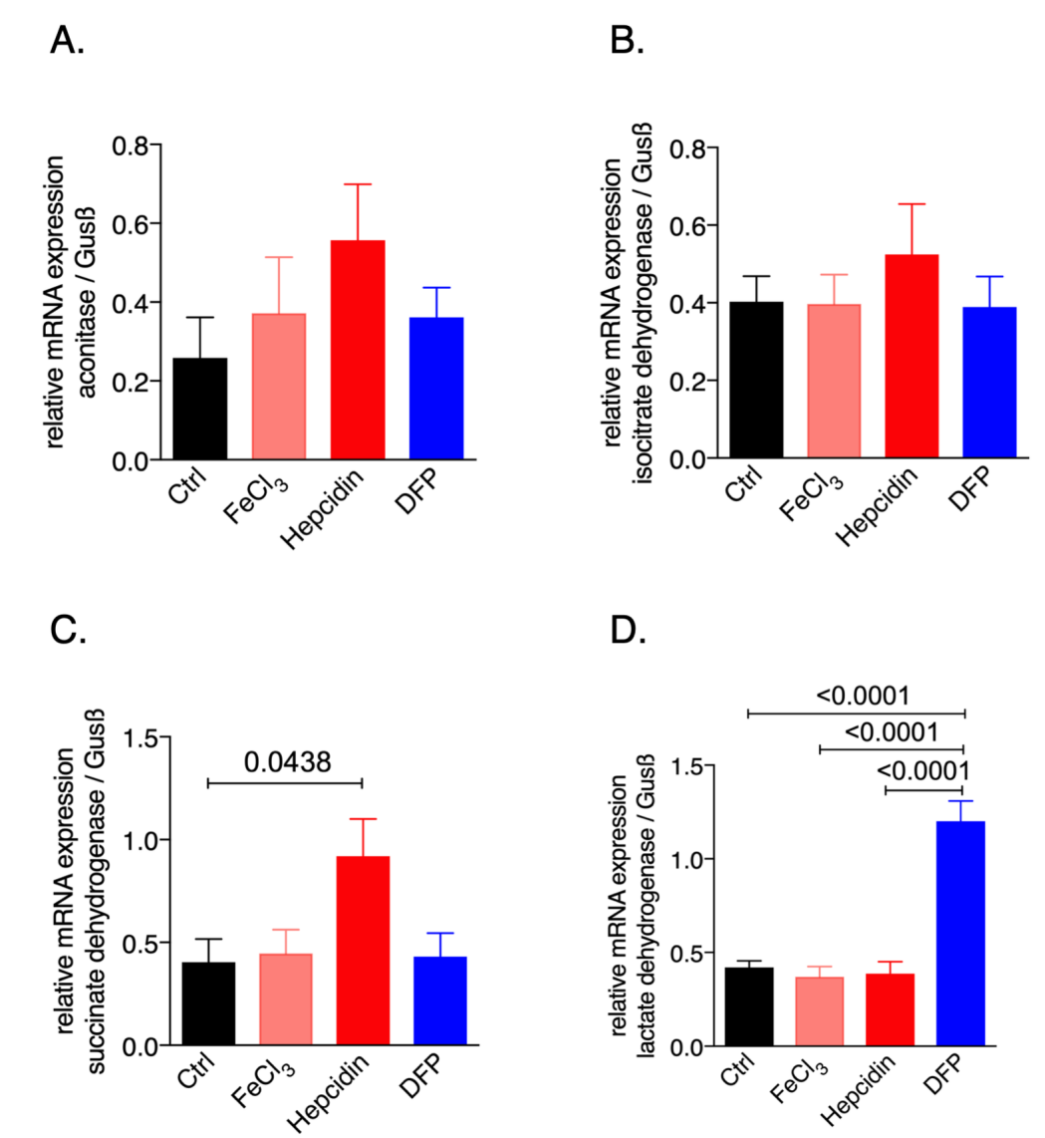
FIGURE 1: Effects of macrophage iron loading on mRNA expression of metabolic enzymes. Expression of aconitase **(A)**, isocitrate dehydrogenase **(B)**, succinate dehydrogenase **(C)** and lactate dehydrogenase **(D)** mRNA relative to the house keeping gene was determined by qRT-PCR after treating RAW264.7 cells with either FeCl_3_ (50 μM), hepcidin (1 μg/ml) or DFP (50 μM) for 30 hours. Representative data from two independent experiments performed with 5 and 6 replicates are shown. Graphs show means ± SEM. One-way ANOVA with Tukey's multiple comparison correction was performed. Exact p-values are indicated in the graphs.

To study the effects of iron availability on macrophage metabolic profiles we used the murine macrophage cell line RAW264.7 and exposed it to iron either by adding ferric chloride or by supplementing hepcidin which blocks iron egress; in addition, to induce iron deficiency the iron chelator deferiprone (DFP) was used. We then examined the mRNA expression pattern of the citric acid enzymes **(Fig. 1)**. While none of the treatments significantly affected the expression of aconitase or isocitrate dehydrogenase (IDH), hepcidin supplementation significantly increased succinate dehydrogenase (SDH, **Fig. 1C)** mRNA expression as compared to the untreated control cells. Instead, iron deficiency following DFP supplementation significantly increased lactate dehydrogenase (LDH) mRNA expression **(Fig. 1D)**. This data provided novel evidence that perturbations of iron homeostasis have an impact on the expression of genes involved in the TCA cycle, thus influencing cellular metabolism and energetics.

### Iron status of macrophages affects the cellular metabolism during an infection

Given the importance of iron homeostasis on cellular energetics, we next investigated the effects of iron perturbations on the regulation of metabolic genes during an infection. In fact, several pathogens including the Gram negative bacterium *Salmonella typhimurium* rely on a sufficient availability of iron for their growth and pathogenicity [33, 34].

**Figure 2:**
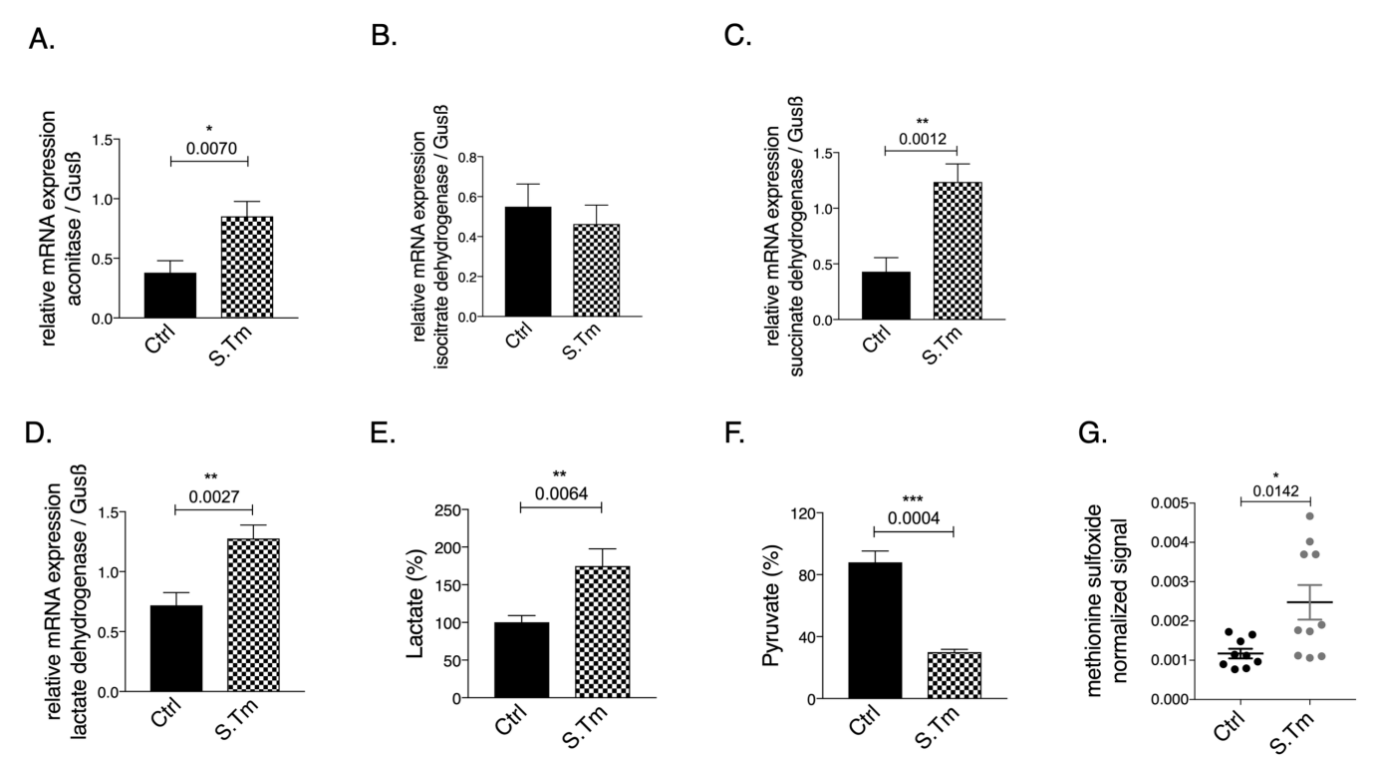
FIGURE 2: Effects of *Salmonella* infection on the expression of metabolic enzymes and metabolites in RAW264.7 macrophages. Cells were infected with *Salmonella* enterica serovar Thyphimurium (MOI of 10) as detailed in methods. Expression of aconitase **(A)**, isocitrate dehydrogenase **(B)**, succinate dehydrogenase **(C)** and lactate dehydrogenase **(D)** mRNA relative to the house keeping gene was determined by qRT-PCR in untreated control cells (ctrl) or infected cells after 24 hours. Pyruvate, lactate and methionine sulfoxide levels **(E-G)** were determined in cell culture supernatants. Representative data from two independent experiments performed with 3 and 6 replicates are shown. Graphs show means ± SEM. Student's t-test was used. Exact p-values are indicated in the graphs.

Thus, we infected RAW264.7 macrophages with *Salmonella* for 24 hours under different iron conditions **(Fig. 2)**. Iron loading of cells was done by combined treatment with hepcidin and ferric chloride because this resembles the situation in iron loaded subjects upon infection where hepcidin is systemically produced and released into the circulation [31, 35].

Cellular infection resulted in significantly increased aconitase **(Fig. 2A**, p=0.007), and SDH **(Fig. 2C**, p=0.0012) mRNA expression, whereas IDH **(Fig. 2B)** mRNA levels remained unchanged compared to uninfected cells.

Of note, infection per se increased the mRNA expression of LDH as compared to uninfected cells **(Fig. 2D**, p=0.0027). In agreement with the Warburg effect, *Salmonella* infection of macrophages resulted in increased lactate **(Fig. 2E**, p=0.0064) and reduced pyruvate concentrations **(Fig. 2F**, p=0.0004) measured in the cellular supernatant as compared to uninfected macrophages, indicating anaerobic glycolysis. Accordingly, metabolomics analysis performed on cellular supernatants indicates changes in cellular metabolites utilization after *Salmonella* infection as compared to macrophages without infection (Suppl. Figure S1). Among the metabolites evaluated, glutamine levels were found to be lower in the supernatant of infected cells suggesting that these cells took up more glutamine compared to controls cells or that less glutamate was released. Glutamine is often used by cells as nutrient and an intermediate to feed the TCA cycle after conversion into glutamate and alpha-ketoglutarate. Moreover, as expected, cells infected with *Salmonella* showed increased oxidative stress, as reflected by the presence of higher levels of the metabolite methionine sulfoxide in the cellular supernatant **(Fig. 2G**, p=0.0142).

**Figure 3:**
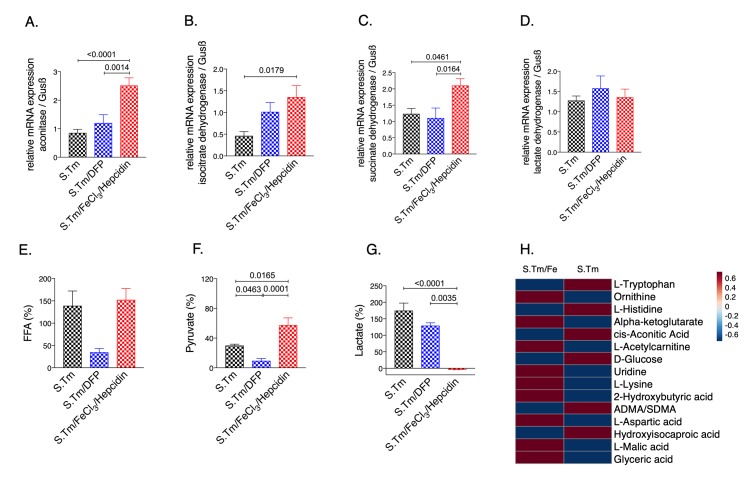
FIGURE 3: Effects of iron perturbations on TCA enzyme expression and metabolite concentrations in *Salmonella* infected macrophages. Cells were pre-treated with FeCl_3_/hepcidin (50 μM/1μg/ml, respectively) or DFP (50 μM) for 6 hours or left untreated prior to infection with *Salmonella* (MOI of 10) as detailed in methods. Expression of aconitase **(A)**, isocitrate dehydrogenase **(B)**, succinate dehydrogenase **(C)** and lactate dehydrogenase **(D)** mRNA relative to the house keeping gene was determined by qRT-PCR in untreated control cells (ctrl) or infected cells after 24 hours. Metabolite levels of lactate **(E)**, free fatty acids **(F)** and pyruvate **(G)** were determined in the cell supernatant after 24 h of infection. A selection of 15-top metabolites (raw-wise) from the metabolomics analysis of cellular supernatants is shown in the heatmap **(H)**, where samples in each group were averaged (column-wise, S.Tm/Fe n=13; S.Tm n=13). Representative data from three independent experiments performed in duplicates or triplicates are shown. Data are shown as relative changes as compared to the control. Graphs show means ± SEM. One-way ANOVA with Tukey's multiple comparison correction was performed. Exact p-values are indicated in the graphs.

Interestingly, iron accumulation in *Salmonella* infected macrophages resulted in significantly higher mRNA expression of aconitase **(Fig. 3A**, p=<0.0001), IDH **(Fig. 3B**, p=0.0179) and SDH **(Fig. 3C**, p=0.0461) as compared to *Salmonella* infected macrophages alone. This translated into elevated pyruvate (Fig. **3F**, p=0.0165) and reduced lactate levels in the culture supernatants **(Fig. 3G**, p=0.0035), indicating reversal of the Warburg effect. Dysregulation of energy metabolism was also captured by spent medium metabolomics analysis (Suppl. Fig. S2). Accordingly, iron supplementation led to an increase in macrophage bacterial load **(Fig. 5E)**. Contrarily, treatment with the iron chelator DFP had only a mild effect on cellular metabolism, rather it significantly reduced *Salmonella* proliferation **(Fig. 3** and **5)** as compared to iron loaded cells.

Overall, these data suggest that activation of anaerobic glycolysis occurs in *Salmonella* infected macrophages, but iron induces a metabolic re-programming leading to an increase in TCA activity with stimulation of TCA enzyme expression.

### Mammalian target of rapamycin (mTOR) signalling pathway is activated during an infection

**Figure 4:**
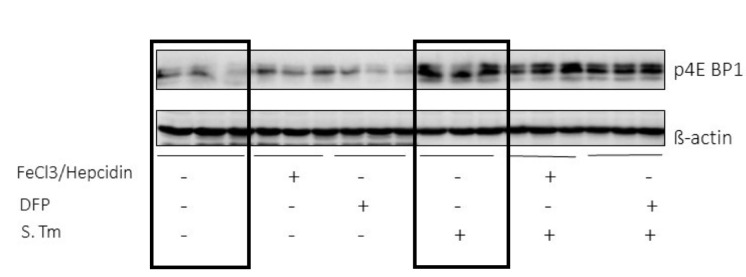
FIGURE 4: Infection but not iron perturbations induces mTOR activation in macrophages. Protein levels of the mTOR downstream target p4E-BP1 were determined using Western blot analysis after treating RAW264.7 cells with *Salmonella* (MOI of 10) for 1 h. Additionally, cells were treated with FeCl3/hepcidin (50 μM/1 μg/ml, respectively) or DFP (50 μM) for 6 h prior to infection.

The data obtained so far indicate iron as an important modulator of the metabolic changes occurring during *Salmonella* infection of macrophages. Based on the description of mTOR as a central regulatory player in sepsis-driven metabolic disturbances [24] we questioned whether the effects of iron perturbations were mediated through the mTOR pathway. So, we determined the phosphorylation of the downstream target of mTOR 4E-BP1 in RAW264.7 macrophages **(Fig. 4)**. We found that infection with *Salmonella* greatly induced 4EBP1 phosphorylation, whereas iron perturbations had little or no effects on this indicator of mTOR activity, neither in un-infected nor in infected macrophages. **(Fig. 4)**.

### Inhibition of mTOR reverses the metabolic reprogramming

**Figure 5:**
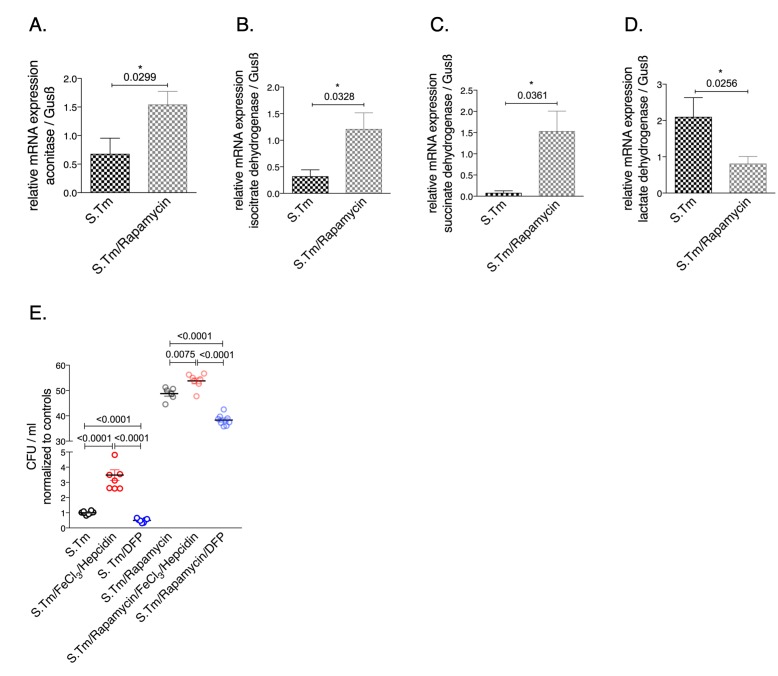
FIGURE 5: Effects of mTOR inhibition on relative expression of metabolic enzymes in *Salmonella* infected RAW264.7 macrophages. Expression of aconitase **(A)**, isocitrate dehydrogenase **(B)**, succinate dehydrogenase **(C)** and lactate dehydrogenase **(D)** mRNA relative to the house keeping gene was determined by qRT-PCR after infecting RAW264.7 cells with *Salmonella* (MOI of 10) for 24 hours and after pre-treatment with rapamycin (200 nM) or saline (control) 1 h prior to infection is shown. The numbers of intramacrophage bacteria was determined by gentamycin protection assay after 24 hours **(E)**. For these experiments, cells were treated with FeCl_3_/hepcidin (50 μM/1 μg/ml, respectively) or DFP (50 μM) for 6 h prior to infection and supplemented with rapamycin (200 nM) or saline one hour before infection. Representative data from three independent experiments performed with duplicates or triplicates are shown. Graphs show means ± SEM. Student's t-test was used. Exact p-values are indicated in the graphs.

To investigate the involvement of the mTOR pathway in the metabolic alterations of macrophages during *Salmonella* infection, infected cells were treated with the mTOR inhibitor rapamycin. Interestingly, we found that rapamycin treatment had an impact on the mRNA levels of citric acid enzymes **(Fig. 5)**. Specifically, mTOR inhibition significantly increased the expression of aconitase, SDH and IDH while it decreased LDH mRNA levels **(Fig. 5)**. Like iron, mTOR affects the expression of critical enzymes in cellular metabolism **(Fig. 5A-D)**. In addition, mTOR inhibition resulted in increased bacterial proliferation **(Fig. 5E)** supporting the importance of mTOR-mediated metabolic reprogramming for the control of infection with the intracellular bacterium *S. typhimurium*. Of note, administration of iron to rapamycin treated macrophages further significantly increased bacterial proliferation whereas DFP reduced it **(Fig. 5)**.

### mTOR signalling pathway impacts iron homeostasis within macrophages

**Figure 6:**
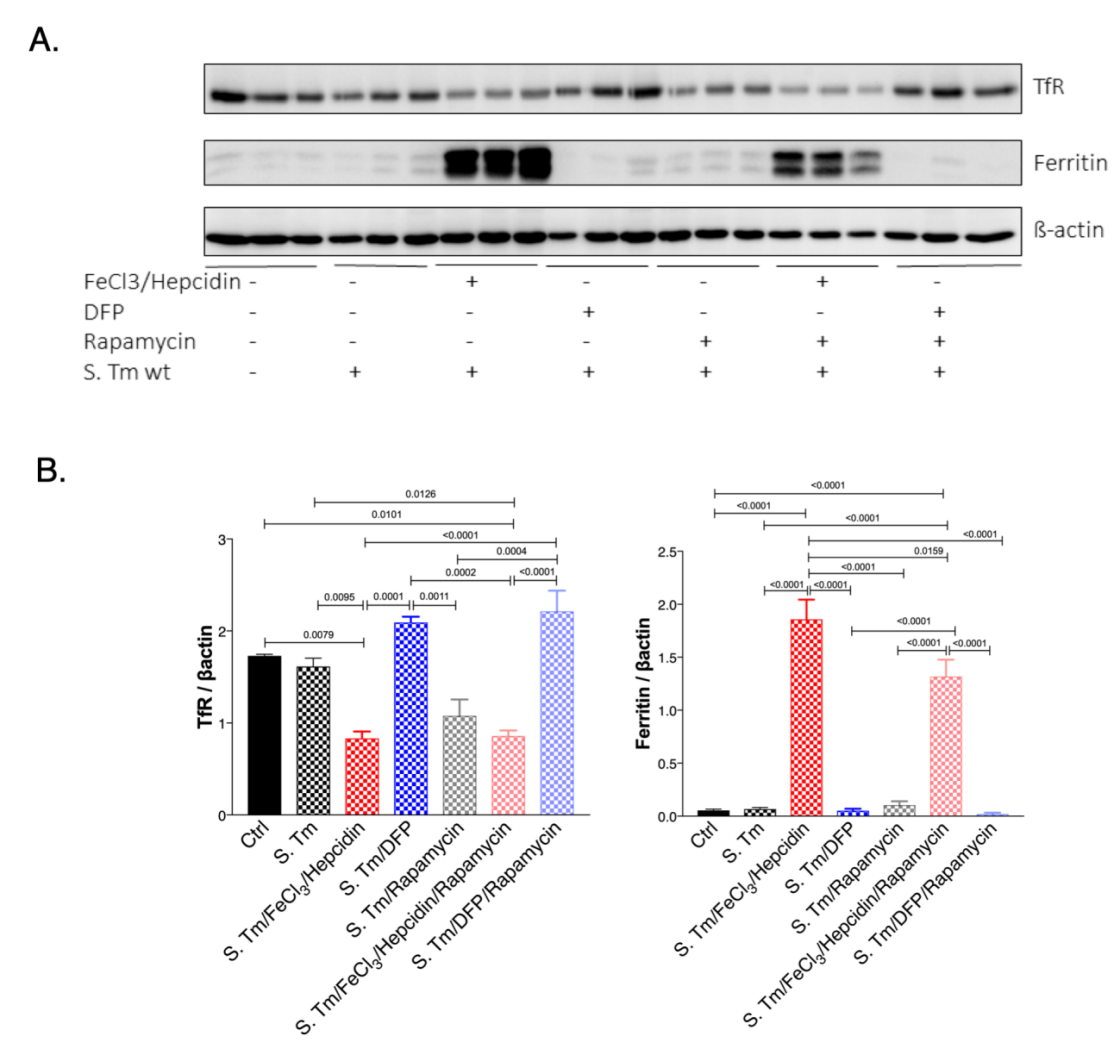
FIGURE 6: Effect of *Salmonella* infection, iron perturbation and mTOR inhibition on iron homeostasis in RAW264.7 cells. Protein levels of transferrin receptor (TfR) and ferritin were determined in control and *Salmonella* infected RAW264.7 cells after 24 hours of infection and either pretreatment with FeCl_3_/hepcidin or DFP and/or rapamycin for 6 or 1 hours respectively. Representative blots **(A)** and densitometric quantification of results relative to beta-actin expression **(B)** are shown. Graphs show means ± SEM. One-way ANOVA with Tukey's multiple comparison correction was performed. Exact p-values (< 0.05) are indicated in the graphs.

Next, we studied if the mTOR signaling pathway could have an impact on iron homeostasis in *Salmonella* infected macrophages **(Fig. 6)**. For this we evaluated the expression of TfR1 which is also a sensitive indicator of intracellular iron availability and because TfR1 has been previously shown to be affected by mTOR activity via TTP [28]. As a marker of iron accumulation in cells we determined ferritin protein levels. While *Salmonella* infection had little effect on TfR and ferritin expression in infected as compared to uninfected macrophages, treatment with iron resulted in the anticipated alterations, namely significant reduction of TfR and increased ferritin expression **(Fig. 6A** and **B)**. DFP treatment had only little effects namely insignificant increase of TfR and decrease of ferritin protein concentrations in this experimental setting. Remarkably, inhibition of mTOR by rapamycin treatment reduced TfR1 protein levels in *Salmonella* infected macrophages. While, rapamycin had no effects on ferritin expression, it blunted the stimulatory effect of iron for ferritin induction indicating that mTOR activity impacts on cellular iron availability by increasing cellular iron accumulation and iron storage within ferritin thereby presumably resulting in reduced levels of metabolically available iron. Conversely, mTOR inhibition by rapamycin would increase intracellular iron availability which may be of benefit for intracellular bacteria. Similar effects of mTOR were also observed in macrophages without infection (Suppl. Fig. S3).

**Figure 7:**
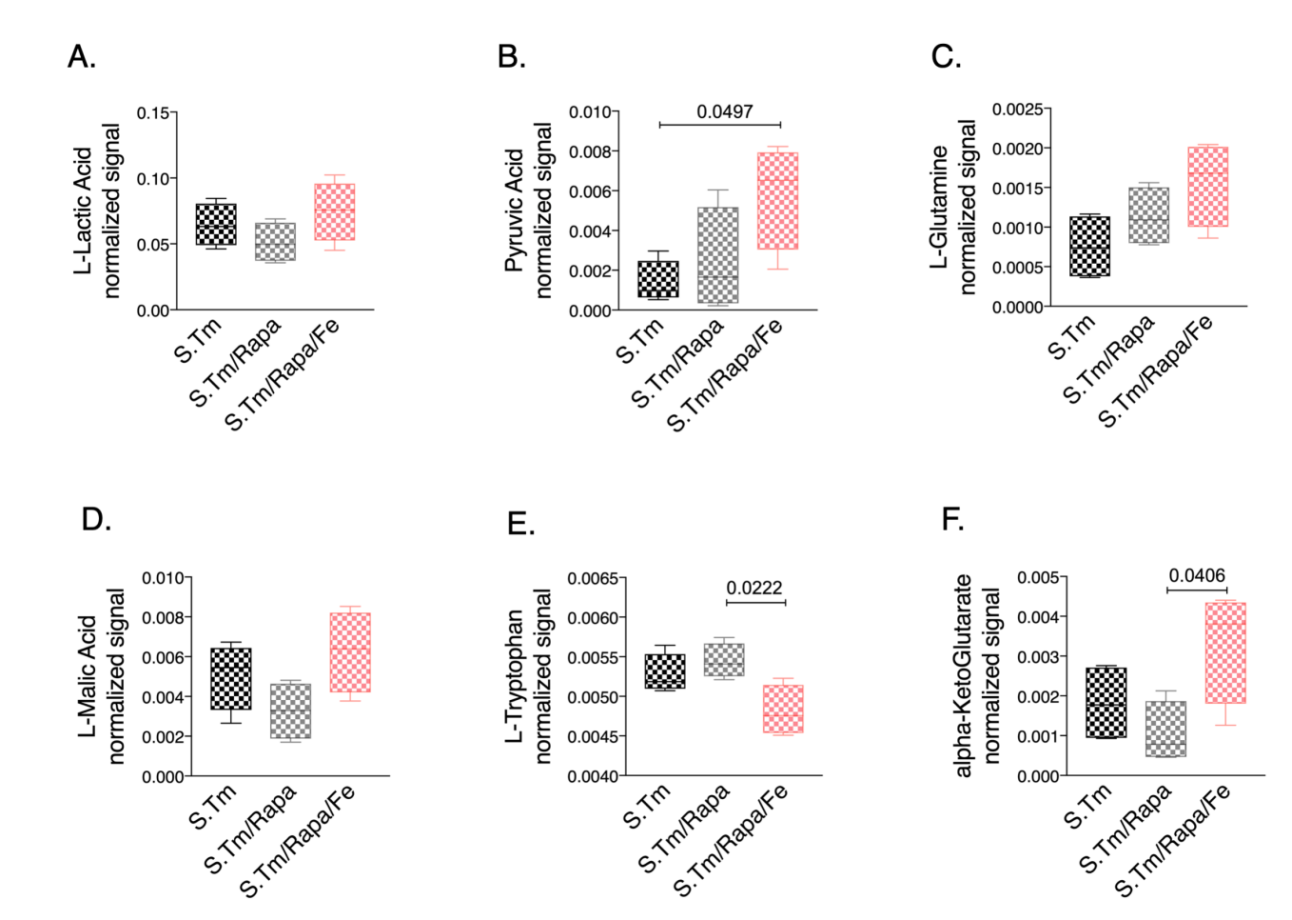
FIGURE 7: Metabolic effect of *Salmonella* infection, iron perturbation and mTOR inhibition in RAW264.7 cells. Selected metabolites annotated after the metabolomics analysis of cell supernatant of cells infected with *Salmonella* and treated with rapamycin or rapamycin and iron/hepcidin (Fe) for 24 hours. Data are shown as box plots. Statistical analysis was performed using One-way ANOVA with Tukey's correction. Exact p-values are indicated in the graphs.

Finally, we studied whether iron treatment also impacted metabolic reprogramming when the mTOR pathway was blocked. Thus, we compared the metabolic profiles in the spent medium of *Salmonella* infected macrophages in the presence and absence of rapamycin with or without iron supplementation **(Fig. 7)**. Of interest, we found that rapamycin treatment had only little effect on lactate and pyruvate concentrations in *Salmonella* infected macrophages. Addition of iron did not significantly change lactate levels but resulted in increased pyruvate concentrations (**Fig. 7A**, **7B**, respectively), whereas iron loading in the absence of rapamycin significantly altered lactate levels as compared to *Salmonella* infected macrophages without additives **(Fig. 3G)**. Moreover, we found increased alpha-ketoglutarate **(Fig. 7F)** and a tendency for increased glutamine **(Fig. 7C)** concentrations in the cellular supernatant of cells treated with rapamycin and iron, suggesting that under such conditions cells are not able to feed the TCA cycle using glutamine as substrate. Accordingly, the malate-aspartate shuttle might also be impaired as malate accumulates in the supernatant **(Fig. 7D)**. In addition, accumulation of ketoleucine indicates that the branched chain amino acid metabolism is affected.

This indicates, that iron may exert part of these metabolic effects by affecting mTOR driven effector pathways specifically by interfering with anaerobic glycolysis and lactate accumulation while stimulating TCA activity. However, we observed several other metabolic pathways which were differently altered by rapamycin treatment and upon concomitant iron loading indicating iron specific effects independent of an influence on the mTOR **(Fig. 7** and Suppl. Fig. S4).

## DISCUSSION

Based on the evidence that both the activation of the mTOR pathway and perturbations of iron homeostasis have subtle effects on different metabolic pathways in cells [1, 4, 36] and because both affect the course of infectious diseases [25, 32], we herein studied their impact on metabolic profiles in *Salmonella* infected macrophages and investigated for a putative functional interaction in that setting. We found that upon *Salmonella* infection metabolic reprogramming characterized by a well described Warburg effect occurs as reflected by induction of aerobic glycolysis [24]. Accordingly, in *Salmonella* infected cells increased lactate and reduced pyruvate levels were found while glutamine and alpha-ketoglutarate concentrations were reduced in cellular supernatants. Glutamine, which is present in culture medium can be consumed by cells and converted to glutamate by glutaminase, which then enter in the TCA cycle upon conversion to alpha-ketoglutarate. This could suggest that upon stimulation of anaerobic glycolysis in the course of infection the cells switch to the use of glutamine to feed TCA cycle.

In addition, iron loading of infected RAW264.7 murine macrophages resulted in induction of TCA activity and reduction of anaerobic glycolysis as evidenced by increased pyruvate and reduced lactate levels. Most interestingly, we provide additional novel information that iron perturbations do not only affect enzymatic activities but also impact on the mRNA expression of the TCA enzymes aconitase (Aco), isocitrate dehydrogenase (IDH) and succinate dehydrogenase (SDH), however, these effects have been slightly different between resting and infected macrophages **(Fig. 1** and **2)**. An effect of iron on TCA activity has so far been mainly attributed to translational regulation of mitochondrial aconitase expression via IRE/IRP interaction [37] but also referred to a direct effect of the metal on enzymatic activities of TCA enzymes as shown in different cellular systems [9, 11]. This may be linked to alterations in the concentrations of enzymatic substrates or a direct impact on the catalytic centers of these enzymes which often contain iron-sulfur clusters [38]. Moreover, the mechanisms by which iron alters the expression of TCA enzymes deserved further analysis of the underlying mechanism but may include epigenetic control by TCA derived carbohydrates and posttranslational regulation of enzyme activities. Of note, we also found that *Salmonella* infection per se resulted in alterations of mRNA expression of some of the TCA enzymes investigated indicating that either innate immune effector molecules or the pathogen by itself impact on their expression and thus metabolic alterations [19, 39, 40]. However, some of these effects could be blocked by the addition of rapamycin indicating that mTOR mediated mechanisms play a central role in regulation of TCA enzymes in the course of infection which may be, however, also affected by the pathogen [41, 42].

Nonetheless, iron loading of macrophages resulted in metabolic programming of macrophages and altered expression of metabolic enzymes. To better understand these alterations, we looked at the activity of the mTOR signaling pathway since prior studies revealed that mTOR may drive the shift towards aerobic glycolysis in acute inflammation [25]. Our results are in line with activation of mTOR pathway in the course of *Salmonella* infection of macrophages as reflected by increased phosphorylation of the downstream target of mTOR, 4E-BP1. In addition, inhibition of mTOR by rapamycin resulted in higher bacterial numbers further supporting the role of mTOR in the control of bacterial infection [26, 43]. However, iron accumulation caused metabolic re-programming but had no direct effect on mTOR activity as evidenced by unaltered phosphorylation of 4E-BP1 **(Fig. 4)**. This would imply that iron availability controls another target of mTOR activity which translates into the Warburg effect. In this context, hypoxia inducible factor 1 (HIF1) has attracted interest as HIF1 activation is centrally involved in transmitting the metabolic effects mediated by the mTOR pathway [24]. On the other hand, the stability of HIF-1 is controlled by iron via its regulatory effect on prolyl-hydroxylases in a way that high iron availability promotes prolyl-hydroxylase activity which then degrades HIF1 [44, 45]. Given the dominant regulatory role of iron for metabolic regulation of infected macrophages it is not surprising that mTOR affects iron in order to limit iron availability and to reduce metabolic reprograming effects. Previous data demonstrated that the tandem zinc finger protein TTP, which is a downstream target of mTOR gets activated in response to rapamycin under low iron conditions or in the presence of an iron chelator. Thereby, TTP negatively regulates the expression of TfR1, leading to reduced iron import [28]. Accordingly, we found a reduction of TfR1 protein expression and reduced ferritin levels upon iron challenge in *Salmonella* infected macrophages treated with rapamycin. This would indicate that mTOR aims at increasing cellular iron acquisition and efficient storage of the metal within ferritin in order to reduce the proportion of metabolically active iron within the cell. Of interest, we also found that upon inhibition of the mTOR pathway by rapamycin in *Salmonella* infected macrophage bacterial numbers significantly increased and metabolic profiles partially changed, however, surprisingly with only little effect on pyruvate and lactate levels. Of note, iron administration to rapamycin treated macrophages further increased bacterial numbers which could on the one hand be due to the fact that mTOR inhibition increased intracellular iron availably for bacteria which serves as a microbial nutrient and that iron inducible metabolic effects result in a pathogen friendly intracellular environment **(Fig. 7)**. Most strikingly, under these circumstances iron induces metabolic reprogramming. Of note, the combined treatment of *Salmonella* infected macrophages with rapamycin and iron resulted in the accumulation of several TCA and branched chain amino acid metabolites. Specifically, the increased concentrations of glutamine and alpha-ketoglutarate as well as of malate in the supernatant suggest problems of feeding the TCA cycle under these combined conditions. The reasons for this remain elusive but may include counter-regulatory mechanisms on enzymatic pathways as well as alterations of mitochondrial function and oxidative stress responses and in addition, effects of the pathogen on metabolic profiles of host cells have also to be considered [46].

One limitation of our study is attributed to the fact that we only investigated RAW 264.7 cells, a well-established model to study macrophage biology and intracellular infection. However, we did not validate those findings in murine primary cells or macrophages isolated from infected mice thus far, an issue with must be followed up in future.

**Figure 8:**
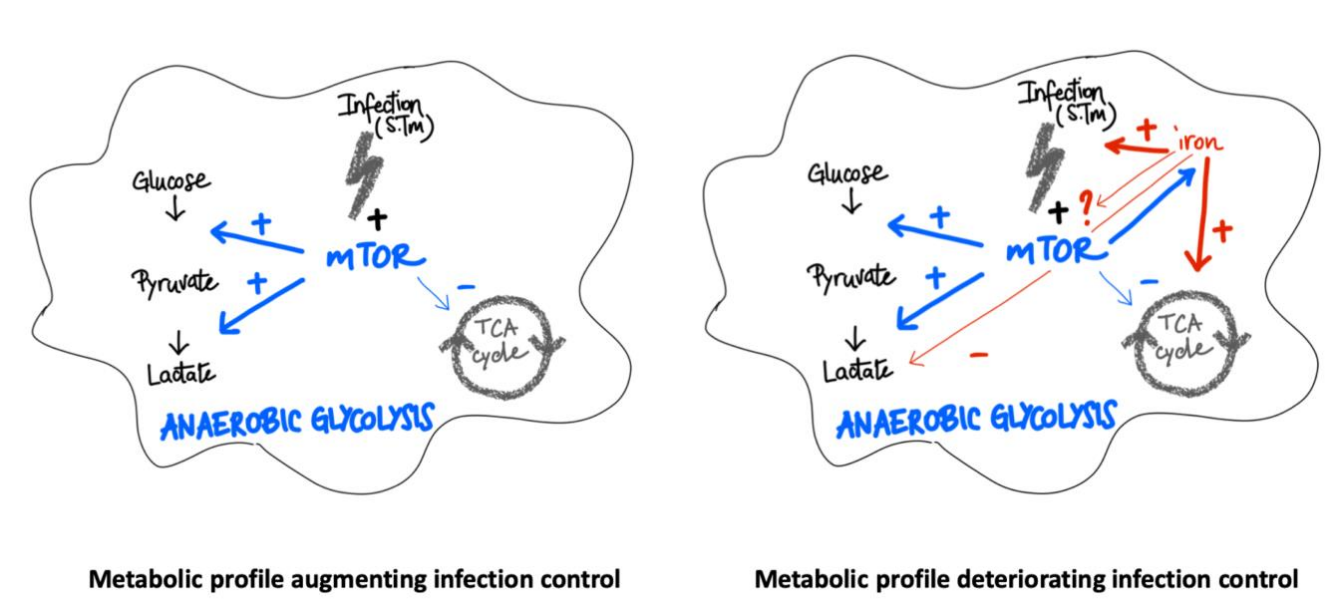
FIGURE 8: Scheme of interactions between iron, mTOR and metabolic activity in macrophages upon *Salmonella* infection. **Left panel:** Upon infection mTOR stimulates the Warburg pathway causing promotion of anaerobic glycolysis whereas citric acid cycle activity (TCA) is reduced resulting in improved control of infection with intracellular bacteria such as *Salmonella*. **Right panel:** Increased abundance of iron in cells activates the TCA cycle but reduces anaerobic glycolysis resulting in a presumably pathogen friendly nutritional environment. In addition, iron is an essential nutrient for *Salmonella* and may feed bacteria directly. To which extend iron and mTOR interact directly or indirectly remains to be shown although blockage of the mTOR pathway even further stimulated the growth promoting effects of iron on *Salmonella*.

In summary, our data indicate that both, the mTOR pathway and iron perturbations have central regulatory effects on metabolic pathways in the course of infection of macrophages with the intracellular bacterium *Salmonella* enterica serovar Typhimurium (S. Tm) **(Fig. 8)**. While mTOR activation induces the Warburg effect and anaerobic glycolysis, iron can cause metabolic re-programming toward TCA activation and aerobic glycolysis which may be partly referred to interference with mTOR inducible metabolic processes. Of note, mTOR function and iron homeostasis are functionally connected with mTOR impacting on cellular iron availability. In addition, iron loading of macrophages affects additional metabolic pathways even when mTOR activity is blocked. Thus, iron may not only act as a primary growth factor for bacteria but also create a microbe friendly metabolic environment within cells. Further studies should investigate the importance of this finding in animal models of infection and further characterize the molecular and metabolic interactions between iron and mTOR and their importance for the control of infectious disease by the host [16, 17, 40].

## MATERIALS AND METHODS

### Cell culture

RAW264.7 murine macrophage-like cells were cultured in complete DMEM supplemented with 10% heat-inactivated fetal calf serum (FCS), 1% penicillin, 1% streptomycin and 1% L-glutamine, 1% minimum essential medium non-essential amino acids (MEM NEAA 100 x) and 2% sodium pyruvate at 37°C in humidified air containing 5% CO_2_.

For simulating an iron overload state, cells were treated with both, FeCl_3_ (50 μM) and hepcidin (Peptanova, Sandhausen, Germany (1 μg/ml) for either 30 hours and on the other hand, to generate iron deficiency within cells, RAW264.7 macrophages were treated with the iron-chelating agent deferiprone (DFP) (50 μM) for 30 hours. Additionally, cells were treated with the mTORC1 inhibitor rapamycin (200 nM) for 1 hour and 20 nM for another 24 hours. Control samples were left untreated. Thereafter, supernatants were collected and stored at -80°C and macrophages were subjected to RNA extraction or protein preparation.

### *Salmonella* infection *in vitro*

Prior to infection, macrophages were incubated in complete medium without antibiotics and pre-stimulated with either FeCl_3_ (50 μM) and hepcidin (1 μg/ml) or DFP (50 μM) for 6 hours. Or rapamycin (200 nM) was supplied 1 hour prior to infection.

Thereafter, the supernatant was removed, and fresh medium was added, supplemented with the same components as described prior with the only difference that only 20 nM of rapamycin was added for subsequent infection period.

Cells were then infected with the viable wild type *Salmonella* enterica serovar Typhimurium (S. Typhimurium, S. Tm wt) strain ATCC14028 for 1 hour at a MOI (multiplicity of infection) of 10 exactly as described elsewhere [46]. The number of *Salmonella* colony forming units (CFU) recovered from macrophages was determined by plating serial dilutions of S. Tm on agar-plates and incubation for 24 hours at 37°C.

### RNA preparation, reverse transcription and polymerase chain reaction

RNA extraction, cDNA transcription and quantitative RT-PCR was performed exactly as described previously [47]. cDNA levels of the respective genes were calculated as relative expression to the house keeping gene beta-Glucuronidase (Gusbeta). The specific probes and primers are presented as supplemental information.

### Western blot analysis

Protein extracts were prepared with cytoplasmic lysis buffer (25 mM Tris–HCl [pH 7.4], 40 mM KCl, and 1% Triton X-100) supplemented with 1 mg/ml aprotinin and 1 mg/ml leupeptin (all obtained from Sigma). 20 μg of total protein were run on 10–15% SDS-polyacrylamide gels, and western blotting was performed using the PVDF transfer membrane as previously described [48]. We used either a mouse anti-human TfR1 antibody (1:1000; Invitrogen, rabbit anti-human ferritin (1:500, Sigma) or rabbit anti-mouse p4E-BP1 (1:1000; Cellsignal) antibodies. Blotting with rabbit anti-β-Actin antibody (1:500; Sigma-Aldrich) was performed as a loading control. The chemiluminescence signal was detected with a ChemiDoc Imaging system (BioRad) and densitometric scanning of blots was carried out for quantitative comparisons.

### Metabolomics analysis of cell supernatants

Cell supernatant samples were collected and stored at -80°C until metabolomics analysis. Samples were diluted adding 200 μL of dilution solution to 50 μL of cell supernatant. The dilution solution consisted of acetonitrile (100%) and contained the following internal standards: alanine-^13^C_3_,^15^N (0.9 μg/mL), arginine- ^13^C_6_,^15^N_4_ (1.8 μg/mL), aspartic acid-^13^C_4_,^15^N (1.3 μg/mL), cystine-^13^C_6_,^15^N_2_ (1.2 μg/mL), glutamic acid-^13^C_5_,^15^N (1.5 μg/mL), glycine-^13^C_2_,^15^N (0.8 μg/mL), histidine-^13^C_6_,^15^N_3_ (1.6 μg/mL), isoleucine-^13^C_6_,^15^N (1.3 μg/mL), leucine-^13^C_6_,^15^N (1.3 μg/mL), lysine-^13^C_6_,^15^N_2_ (1.5 μg/mL), methionine-^13^C_5_,^15^N (1.5 μg/mL), phenylalanine-^13^C_9_,^15^N (1.7 μg/mL), proline-^13^C_5_,^15^N (1.2 μg/mL), serine-^13^C_3_,^15^N (1.1 μg/mL), threonine-^13^C_4_,^15^N (1.2 μg/mL), tyrosine-^13^C_9_,^15^N (1.8 μg/mL), and valine-^13^C_5_,^15^N (1.2 μg/mL). Samples were then filtered through a protein removal plate (Sirocco, Waters Corporation, Milford, MA, USA). Quality control (QC) samples were obtained by pooling together small aliquots (15 μL) from each sample.

### Ultra-high-performance liquid chromatography (UHPLC) combined with Mass spectrometry (MS)

All samples were analyzed using a metabolomics workflow previously described [49]. Briefly, ultra-high-performance liquid chromatography (UHPLC) (Agilent 1290; Agilent Technologies, Santa Clara, CA, USA) was coupled to a Q-TOF mass spectrometer (TripleTOF 5600+; AB Sciex, Foster City, CA, USA). The chromatographic separation was achieved by hydrophilic interaction liquid chromatography (HILIC) using an Acquity BEH amide, 100 × 2.1 mm column (Waters Corporation, Milford, MA, USA).

Acetonitrile + 0.1% formic acid was used as mobile phase A and water + 0.1% formic acid as mobile phase B. The injection volume was set at 5 μl, and the flow rate at 0.6 ml/min. The following linear gradients were used: 0 min 95% A and 1 min 95% A, 4 min 30% A and 5 min 30% A, and 5.1 min 95% A and 8 min 95% A.

The mass spectrometer operated in full scan mode in the mass range from 50 to 1000 m/z and with an accumulation time of 250 ms. In ESI+ mode, the source temperature was set at 700°C, the declustering potential at 30 V, the collision energy at 6 V, the ion spray voltage at 5120 V, the curtain gas at 25 psi, and the ion source gases 1 and 2 at 60 psi. In ESI+ mode, the source temperature was set at 650°C, the declustering potential at − 45 V, the collision energy at − 6 V, the ion spray voltage at − 3800 V, the curtain gas at 25 psi, and the ion source gases 1 and 2 at 30 psi. The instrument was mass calibrated by automatic calibration infusing the Sciex Positive Calibration Solution (part no. 4460131, AB Sciex, Foster City, CA, USA) for positive mode and Sciex Negative Calibration Solution (part no. 4460134, AB Sciex, Forster City, CA, USA) for negative mode after every two sample injections. Samples were then analyzed in randomized order, and pooled QC samples were injected every eight samples.

The identification of metabolites was obtained by verifying retention time, accurate mass, and tandem mass spectrometry data against our in-house and/or online databases, including the Human Metabolome Database (HMDB) [50] and the METLIN database [51]. For the analysis, metabolomics data were normalized by the sum of the features, then log transformed and unit variance scaled using MetaboAnalyst (version 4.0) [52]. Heatmaps were generated using MetaboAnalyst.

### Statistical analysis

Significance was determined by analysis of variance (ANOVA) combined with Tukey's correction. Additionally, Student's t-test was used for comparing two different groups. Generally, P values less than 0.05 were considered significant in any test.

## SUPPLEMENTAL MATERIAL

Click here for supplemental data file.

All supplemental data for this article are available online at www.microbialcell.com/researcharticles/2019a-telser-microbial-cell/.

## Abbreviations:

DFP: – deferiprone,
DMT-1: – divalent metal transporter 1,
HIF1: – hypoxia inducible factor 1,
IDH: – isocitrate dehydrogenase,
IRE: – iron responsive element,
IRP: – iron regulatory protein,
LDH: – lactate dehydrogenase,
mTOR: – mammalian target of rapamycin,
SDH: – succinate dehydrogenase,
TCA: – tricarboxylic acid cycle,
TfR: – transferrin receptor,
TTP: – tristetraprolin.

## References

[1] Muckenthaler MU, Rivella S, Hentze MW, Galy B (2017). A Red Carpet for Iron Metabolism.. Cell.

[2] Koskenkorva-Frank TS, Weiss G, Koppenol WH, Burckhardt S (2013). The complex interplay of iron metabolism, reactive oxygen species, and reactive nitrogen species: Insights into the potential of various iron therapies to induce oxidative and nitrosative stress.. Free Radic Biol Med.

[3] Nairz M, Theurl I, Swirski FK, Weiss G (2017). Pumping iron"-how macrophages handle iron at the systemic, microenvironmental, and cellular levels.. Pflugers Arch.

[4] Pantopoulos K, Porwal SK, Tartakoff A, Devireddy L (2012). Mechanisms of Mammalian Iron Homeostasis.. Biochemistry.

[5] Rouault TA, Maio N (2017). Biogenesis and functions of mammalian iron-sulfur proteins in the regulation of iron homeostasis and pivotal metabolic pathways.. J Biol Chem.

[6] Ruiz JC, Walker SD, Anderson SA, Eisenstein RS, Bruick RK (2013). F-box and leucine-rich repeat protein 5 (FBXL5) is required for maintenance of cellular and systemic iron homeostasis.. J Biol Chem.

[7] Kim H-Y, LaVaute T, Iwai K, Klausner RD, Rouault TA (1996). Identification of a Conserved and Functional Iron-responsive Element in the 5′-Untranslated Region of Mammalian Mitochondrial Aconitase.. J Biol Chem.

[8] Schalinske KL, Chen OS, Eisenstein RS (1998). Iron Differentially Stimulates Translation of Mitochondrial Aconitase and Ferritin mRNAs in Mammalian Cells.. J Biol Chem.

[9] Oexle H, Gnaiger E, Weiss G (1999). Iron-dependent changes in cellular energy metabolism: influence on citric acid cycle and oxidative phosphorylation.. Biochim Biophys Acta.

[10] Ross KL, Eisenstein RS (2002). Iron Deficiency Decreases Mitochondrial Aconitase Abundance and Citrate Concentration without Affecting Tricarboxylic Acid Cycle Capacity in Rat Liver.. J Nutr.

[11] Volani C, Paglia G, Smarason S, Pramstaller P, Demetz E, Pfeifhofer-Obermair C, Weiss G (2018). Metabolic Signature of Dietary Iron Overload in a Mouse Model.. Cells.

[12] Volani C, Doerrier C, Demetz E, Haschka D, Paglia G, Lavdas AA, Gnaiger E, Weiss G (2017). Dietary iron loading negatively affects liver mitochondrial function.. Metallomics.

[13] Girelli D, Nemeth E, Swinkels DW (2016). Hepcidin in the diagnosis of iron disorders.. Blood.

[14] Nemeth E, Tuttle MS, Powelson J, Vaughn MB, Donovan A, Ward DM, Ganz T, Kaplan J (2004). Hepcidin Regulates Cellular Iron Efflux by Binding to Ferroportin and Inducing Its Internalization.. Science.

[15] Theurl I, Aigner E, Theurl M, Nairz M, Seifert M, Schroll A, Sonnweber T, Eberwein L, Witcher DR, Murphy AT, Wroblewski VJ, Wurz E, Datz C, Weiss G (2009). Regulation of iron homeostasis in anemia of chronic disease and iron deficiency anemia: diagnostic and therapeutic implications.. Blood.

[16] Soares MP, Weiss G (2015). The Iron age of host-microbe interactions.. EMBO Rep.

[17] Skaar EP, Raffatellu M (2015). Metals in infectious diseases and nutritional immunity.. Metallomics.

[18] Porto G, De Sousa M (2007). Iron overload and immunity.. World J Gastroenterol.

[19] Weiss G, Schaible UE (2015). Macrophage defense mechanisms against intracellular bacteria.. Immunol Rev.

[20] Stefanova D, Raychev A, Arezes J, Ruchala P, Gabayan V, Skurnik M, Dillon BJ, Horwitz MA, Ganz T, Bulut Y, Nemeth E (2017). Endogenous hepcidin and its agonist mediate resistance to selected infections by clearing non–transferrin-bound iron.. Blood.

[21] Nairz M, Ferring-Appel D, Casarrubea D, Sonnweber T, Viatte L, Schroll A, Haschka D, Fang FC, Hentze MW, Weiss G, Galy B (2015). Iron Regulatory Proteins Mediate Host Resistance to Salmonella Infection.. Cell Host Microbe.

[22] Drakesmith H, Prentice AM (2012). Hepcidin and the iron-infection axis.. Science.

[23] Vander Heiden MG, Cantley LC, Thompson CB (2009). Understanding the Warburg effect: the metabolic requirements of cell proliferation.. Science.

[24] Cheng S-C, Quintin J, Cramer RA, Shepardson KM, Saeed S, Kumar V, Giamarellos-Bourboulis EJ, Martens JHA, Rao NA, Aghajanirefah A, Manjeri GR, Li Y, Ifrim DC, Arts RJW, van der Veer BMJW, Deen PMT, Logie C, O'Neill LA, Willems P, van de Veerdonk FL, van der Meer JWM, Ng A, Joosten LAB, Wijmenga C, Stunnenberg HG, Xavier RJ, Netea MG, Netea MG (2014). mTOR- and HIF-1-mediated aerobic glycolysis as metabolic basis for trained immunity.. Science.

[25] Cheng S-C, Scicluna BP, Arts RJW, Gresnigt MS, Lachmandas E, Giamarellos-Bourboulis EJ, Kox M, Manjeri GR, Wagenaars JAL, Cremer OL, Leentjens J, van der Meer AJ, van de Veerdonk FL, Bonten MJ, Schultz MJ, Willems PHGM, Pickkers P, Joosten LAB, van der Poll T, Netea MG (2016). Broad defects in the energy metabolism of leukocytes underlie immunoparalysis in sepsis.. Nat Immunol.

[26] Lachmandas E, Beigier-Bompadre M, Cheng S-C, Kumar V, van Laarhoven A, Wang X, Ammerdorffer A, Boutens L, de Jong D, Kanneganti T-D, Gresnigt MS, Ottenhoff THM, Joosten LAB, Stienstra R, Wijmenga C, Kaufmann SHE, van Crevel R, Netea MG (2016). Rewiring cellular metabolism via the AKT/mTOR pathway contributes to host defence against Mycobacterium tuberculosis in human and murine cells.. Eur J Immunol.

[27] Mleczko-Sanecka K, Roche F, da Silva AR, Call D, D'Alessio F, Ragab A, Lapinski PE, Ummanni R, Korf U, Oakes C, Damm G, D'Alessandro LA, Klingmüller U, King PD, Boutros M, Hentze MW, Muckenthaler MU (2014). Unbiased RNAi screen for hepcidin regulators links hepcidin suppression to proliferative Ras/RAF and nutrient-dependent mTOR signaling.. Blood.

[28] Bayeva M, Khechaduri A, Puig S, Chang H-C, Patial S, Blackshear PJ, Ardehali H (2012). mTOR Regulates Cellular Iron Homeostasis through Tristetraprolin.. Cell Metab.

[29] Guan P, Wang N (2014). Mammalian target of rapamycin coordinates iron metabolism with iron-sulfur cluster assembly enzyme and tristetraprolin.. Nutrition.

[30] Sato T, Chang H-C, Bayeva M, Shapiro JS, Ramos-Alonso L, Kouzu H, Jiang X, Liu T, Yar S, Sawicki KT, Chen C, Martínez-Pastor MT, Stumpo DJ, Schumacker PT, Blackshear PJ, Ben-Sahra I, Puig S, Ardehali H (2018). mRNA-binding protein tristetraprolin is essential for cardiac response to iron deficiency by regulating mitochondrial function.. Proc Natl Acad Sci U S A.

[31] Nairz M, Schroll A, Haschka D, Dichtl S, Tymoszuk P, Demetz E, Moser P, Haas H, Fang FC, Theurl I, Weiss G (2017). Genetic and Dietary Iron Overload Differentially Affect the Course ofSalmonellaTyphimurium Infection.. Front Cell Infect Microbiol.

[32] Weiss G, Carver PL (2018). Role of divalent metals in infectious disease susceptibility and outcome.. Clin Microbiol Infect.

[33] Bumann D, Schothorst J (2017). Intracellular Salmonella metabolism.. Cell Microbiol.

[34] Andrews-Polymenis HL, Baumler AJ, McCormick BA, Fang FC (2010). Taming the Elephant: Salmonella Biology, Pathogenesis, and Prevention.. Infect Immun.

[35] Ganz T, Nemeth E (2015). Iron homeostasis in host defence and inflammation.. Nat Rev Immunol.

[36] Kim J, Guan K-L (2019). mTOR as a central hub of nutrient signalling and cell growth.. Nat Cell Biol.

[37] Galy B, Ferring-Appel D, Sauer SW, Kaden S, Lyoumi S, Puy H, Kölker S, Gröne H-J, Hentze MW (2010). Iron Regulatory Proteins Secure Mitochondrial Iron Sufficiency and Function.. Cell Metab.

[38] Rampelt H, van der Laan M (2017). The Yin & Yang of Mitochondrial Architecture - Interplay of MICOS and F1Fo-ATP synthase in cristae formation.. Microb Cell.

[39] Pearce EJ, Pearce EL (2018). Driving immunity: all roads lead to metabolism.. Nat Rev Immunol.

[40] Ryan DG, Murphy MP, Frezza C, Prag HA, Chouchani ET, O'Neill LA, Mills EL (2019). Coupling Krebs cycle metabolites to signalling in immunity and cancer.. Nat Metab.

[41] Ganesan R, Hos NJ, Gutierrez S, Fischer J, Stepek JM, Daglidu E, Krönke M, Robinson N (2017). Salmonella Typhimurium disrupts Sirt1/AMPK checkpoint control of mTOR to impair autophagy.. PLoS Pathog.

[42] Stienstra R, Netea-Maier RT, Riksen NP, Joosten LAB, Netea MG (2017). Specific and Complex Reprogramming of Cellular Metabolism in Myeloid Cells during Innate Immune Responses.. Cell Metab.

[43] Eiden AM, Zhang S, Gary JM, Simmons JK, Mock BA (2016). Molecular Pathways: Increased Susceptibility to Infection Is a Complication of mTOR Inhibitor Use in Cancer Therapy.. Clin Cancer Res.

[44] Wilkins JM, Trushina E (2017). Application of Metabolomics in Alzheimer's Disease.. Front Neurol.

[45] Haase VH (2017). Therapeutic targeting of the HIF oxygen-sensing pathway: Lessons learned from clinical studies.. Exp Cell Res.

[46] Frawley ER, Crouch M-L, Bingham-Ramos LK, Robbins HF, Wang W, Wright GD, Fang FC (2013). Iron and citrate export by a major facilitator superfamily pump regulates metabolism and stress resistance in Salmonella Typhimurium.. Proc Natl Acad Sci U S A.

[47] Dichtl S, Demetz E, Haschka D, Tymoszuk P, Petzer V, Nairz M, Seifert M, Hoffmann A, Brigo N, Würzner R, Theurl I, Karlinsey JE, Fang FC, Weiss G (2019). Dopamine Is a Siderophore-Like Iron Chelator That Promotes *Salmonella enterica* Serovar Typhimurium Virulence in Mice.. MBio.

[48] Dichtl S, Haschka D, Nairz M, Seifert M, Volani C, Lutz O, Weiss G (2018). Dopamine promotes cellular iron accumulation and oxidative stress responses in macrophages.. Biochem Pharmacol.

[49] Volani C, Caprioli G, Calderisi G, Sigurdsson BB, Rainer J, Gentilini I, Hicks AA, Pramstaller PP, Weiss G, Smarason S V., Paglia G (2017). Pre-analytic evaluation of volumetric absorptive microsampling and integration in a mass spectrometry-based metabolomics workflow.. Anal Bioanal Chem.

[50] Wishart DS, Jewison T, Guo AC, Wilson M, Knox C, Liu Y, Djoumbou Y, Mandal R, Aziat F, Dong E, Bouatra S, Sinelnikov I, Arndt D, Xia J, Liu P, Yallou F, Bjorndahl T, Perez-Pineiro R, Eisner R, Allen F, Neveu V, Greiner R, Scalbert A (2012). HMDB 3.0—The Human Metabolome Database in 2013.. Nucleic Acids Res.

[51] Zhu Z-J, Schultz AW, Wang J, Johnson CH, Yannone SM, Patti GJ, Siuzdak G (2013). Liquid chromatography quadrupole time-of-flight mass spectrometry characterization of metabolites guided by the METLIN database.. Nat Protoc.

[52] Chong J, Soufan O, Li C, Caraus I, Li S, Bourque G, Wishart DS, Xia J (2018). MetaboAnalyst 4.0: towards more transparent and integrative metabolomics analysis.. Nucleic Acids Res.

