# Supplementary material for: Metabolic reprogramming of Salmonella infected macrophages and its modulation by iron availability and the mTOR pathway
Source: Microb Cell. 2019 Nov 14;6(12):531–43. doi: 10.15698/mic2019.12.700 (PMC6883347; doi:10.15698/mic2019.12.700)
Supplement: Supplementary file 1 [file mic-06-531-s01.pdf]

SUPPELEMENTAL MATERIAL

Metabolic reprogramming of *Salmonella* infected macrophages and its modulation by iron availability and the mTOR pathway

Julia Telser[1,2,#], Chiara Volani[1,3,#], Richard Hilbe[1,2], Markus Seifert[1,2], Natascha Brigo[1], Giuseppe Paglia[4] and Günter Weiss[1,2,*]

[1] Department of Internal Medicine II, Medical University of Innsbruck, Austria.
[2] Christian Doppler Laboratory for Iron Metabolism and Anemia Research, Medical University of Innsbruck, Austria.
[3] EURAC Research, Institute for Biomedicine, Bolzano/Bozen, Italy.
[4] School of Medicine and Surgery, Univerity of Milano-Bicocca.
[#] Equal contribution.
* Corresponding Author:
Guenter Weiss, MD, Department of Internal Medicine II, Infectious Diseases, Immunology, Pneumology and Rheumatology, Medical University Innsbruck, Anichstr. 35, A-6020 Innsbruck, Austria; Phone: +43-512-504-23251; Fax: +43-512-504-23317; E-mail: guenter.weiss@i-med.ac.at

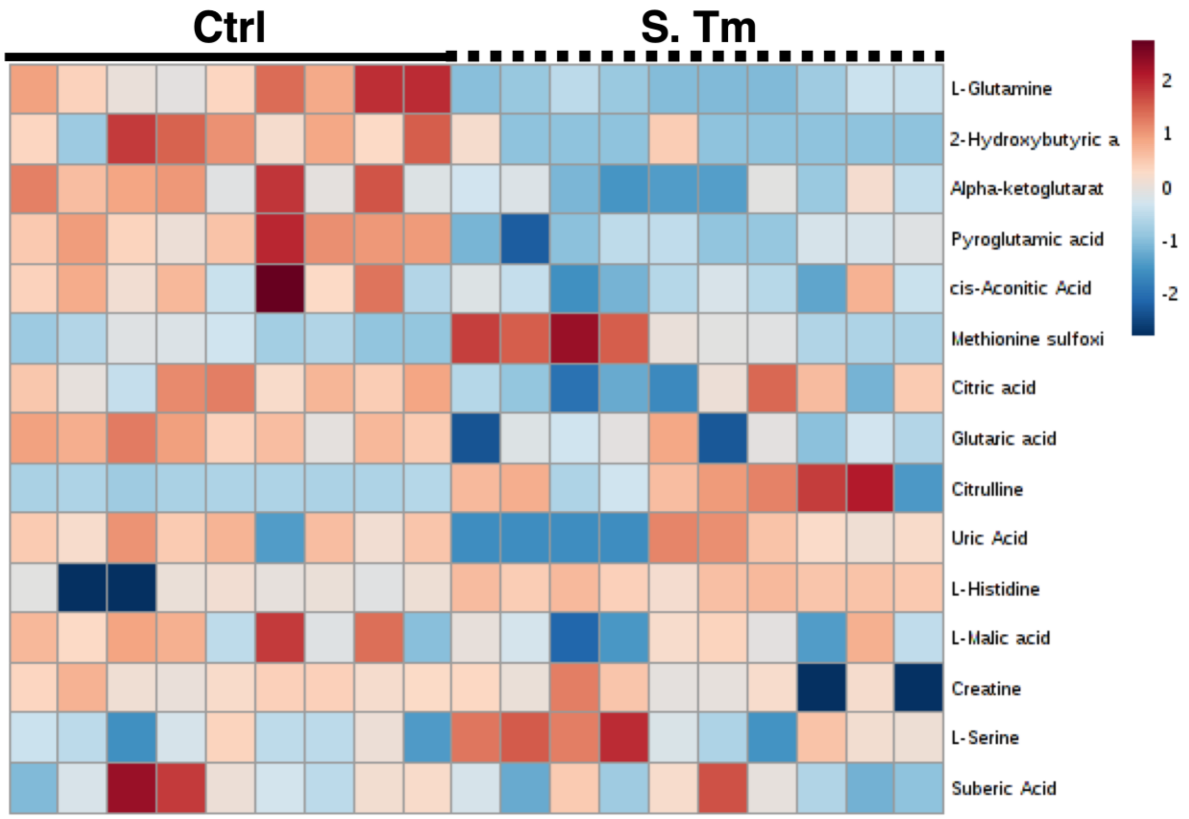

Supplementary Figure S1: *Salmonella* infection leads to a metabolic remodeling of RAW264.7 cells that involved the TCA cycle. Heatmap showing the 15-top metabolites annotated from the metabolomics analysis of the cellular supernatants, that showed the greater changes between control (Ctrl, *black bold line*) and *Salmonella* infected macrophages (S. Tm wt, *black dashed line*). For every single sample (column-wise) several metabolites were analyzed as described in the Material and Method section, 15-top metabolites (raw-wise) were selected from the analysis for the graphical representation.

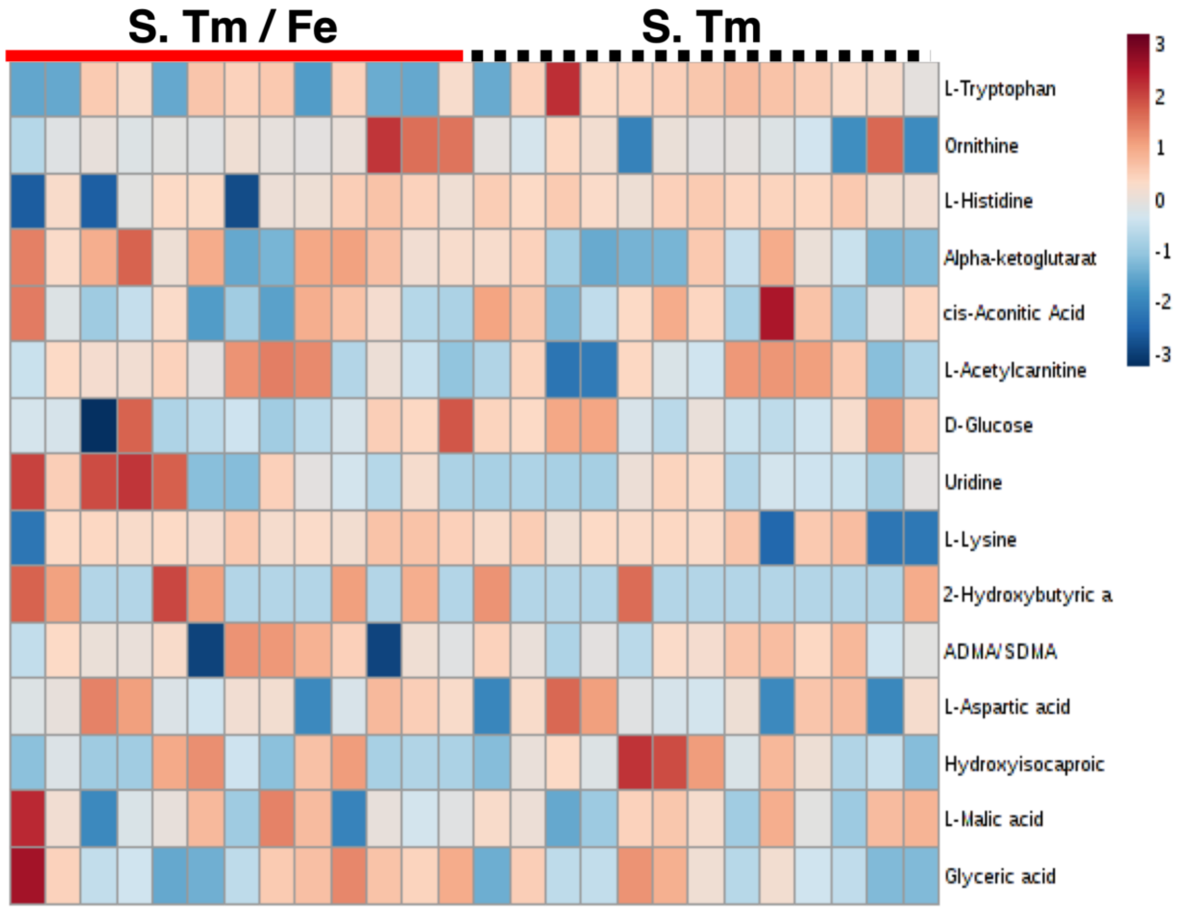

Supplementary Figure S2: Heatmap of metabolic effects of rapamycin with and without iron supplementation in *Salmonella* infected macrophages. Heatmap showing the 15-top metabolites, annotated from the metabolomics analysis of the cellular supernatants, that showed the greater changes between infected macrophages (S.Tm, *black dashed line*) and infected macrophages exposed to iron and hepcidin (S. Tm wt + Fe, *red line*). For every single sample (column-wise) several metabolites were analyzed as described in the Material and Method section, 15-top metabolites (raw-wise) were selected from the analysis for the graphical representation.

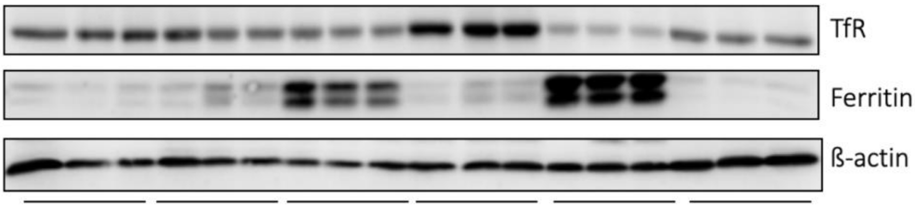

FeCl3/Hepcidin	-	-	+	-	-	-
DFP	-	-	-	+	-	-
Rapamycin	-	+	-	-	-	-
FeCl3/Hepcidin/Rapamycin	-	-	-	-	+	-
DFP/Rapamycin	-	-	-	-	-	+

Supplementary Figure S3: Effects of iron perturbations and rapamycin on the expression of iron homeostasis genes in uninfected RAW264.7 cells. Protein levels of transferrin receptor (TfR) and ferritin were determined in control and *Salmonella* infected RAW264.7 cells after 24 hours of infection and either pretreatment with FeCl3/hepcidin or DFP and/or rapamycin for 6 or 1 hours respectively. A representative blot is shown.

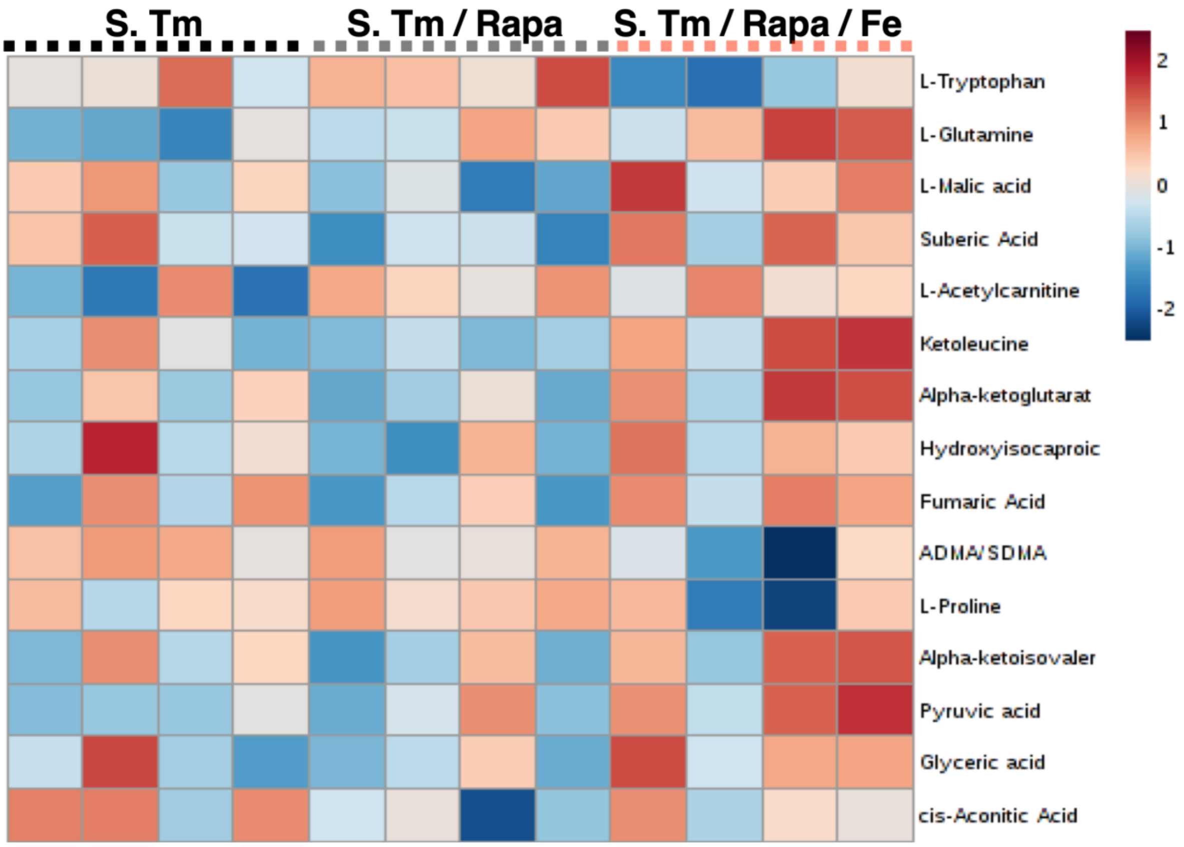

Supplementary Figure S4: Heatmap of metabolic effects of rapamycin with and without iron supplementation in *Salmonella* infected macrophages. Heatmap showing the 15-top metabolites, annotated from the metabolomics analysis of the cellular supernatants, that showed the greater changes comparing infected macrophages (S.Tm wt, *black dashed line*) with infected macrophages with rapamycin (S. Tm wt + Rapa, *grey dashed line*) and with infected macrophages with rapamycin and iron (S.Tm + Rapa + Fe, *pink dashed line)*. Rapa: Rapamycin; Fe: $FeCl_3$/Hepcidin. For every single sample (column-wise) several metabolites were analyzed as described in the Material and Method section, 15-top metabolites (raw-wise) were selected from the analysis for the graphical representation.

Supplementary TABLE S1. Primer and probes.

Primer/Probe	Sequence (5'-3')	Annealing Temp. (°C)
mu_Ldha_fw	GCTCCCCAGAACAAGATTACAG	60°C
mu_Ldha_rv	TCGCCCTTGAGTTTGTCTTC	60°C
mu_Aco2_fw	GCCCTTTACCCCTGACTTG	60°C
mu_Aco2_rv	GTCCCATGTCCTCATAGCTTG	60°C
mu_Idh2_fw	GAAGAGGATCAAGGTGGAGAAG	60°C
mu_Idh2_rv	GGAAGCCCAAGGTCAAAATAC	60°C
mu_Sdhd_fw	TCTCTTAAAGCTGGGCGTTC	60°C
mu_Sdhd_rv	AGGTGAATGTGCTGGGTAC	60°C
mu_Gusß_fw	CTCATCTGGAATTTCGCCGA	60°C
mu_Gusß_rv	GGCGAGTGAAGATCCCCTTC	60°C
mu_Gusß_Probe	CGAACCAGTCACCGCTGAGAGTAATCG	60°C

Abbreviations used: LDHa, lactate dehydrogenaseA; Aco2, Aconitase2, Idh2, Isocitrate Dehyrogenase2; Sdhd, Succinate DehydrogenaseD; Gusß, β-glucuronidase
